# 299. Metagenomic Next-Generation Sequencing of Cerebrospinal Fluid for Central Nervous System Infections: Clinical Factors Associated with Increased Diagnostic Yield

**DOI:** 10.1093/ofid/ofae631.089

**Published:** 2025-01-29

**Authors:** Patrick Benoit, Mikael de Lorenzi-Tognon, Noah Brazer, Emily Kelly, Venice Servellita, Miriam Oseguera, Jenny Nguyen, Jack Tang, Charles Omura, Jessica Streithorst, Melissa Hillberg, Danielle Ingebrigtsen, Chengshi Jin, Ann Lazar, Charles Chiu

**Affiliations:** University of California San Francisco, San Francisco, CA; University of California San Francisco, San Francisco, CA; University of California San Francisco, San Francisco, CA; University of California San Francisco, San Francisco, CA; University of California San Francisco, San Francisco, CA; University of California San Francisco, San Francisco, CA; University of California San Francisco, San Francisco, CA; University of California San Francisco, San Francisco, CA; University of California San Francisco, San Francisco, CA; University of California San Francisco, San Francisco, CA; University of California San Francisco, San Francisco, CA; University of California San Francisco, San Francisco, CA; University of California San Francisco, San Francisco, CA; University of California San Francisco, San Francisco, CA; UCSF, San Francisco, California

## Abstract

**Background:**

Metagenomic next-generation sequencing (mNGS) of cerebrospinal fluid (CSF) can improve diagnostic yields in central nervous system (CNS) infections, but the most beneficial clinical indications have yet to be defined.

**Table 1.**
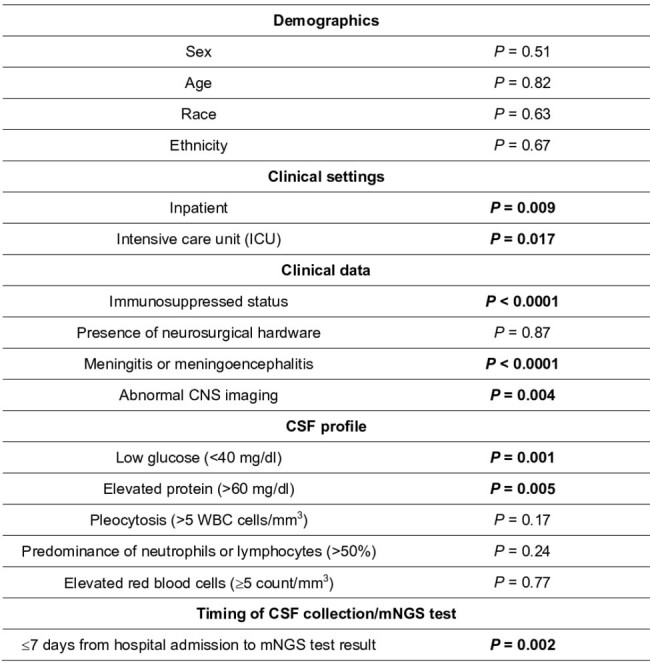
Factors evaluated by univariate analysis associated with a positive CSF mNGS test *P-value in bold are considered statistically significant (P < 0.05)

**Methods:**

We analyzed CSF mNGS results and associated clinical, demographic, and laboratory metadata from a cohort of patients for whom mNGS testing was performed at University of California, San Francisco (UCSF) from 2016 to 2023. We assessed by chart-review positive CSF mNGS results that established definitive diagnoses of CNS infections in the cohort. We then used univariate and multivariate logistic regression analysis to identify clinical factors that were associated with positive mNGS results.

**Figure 1.**
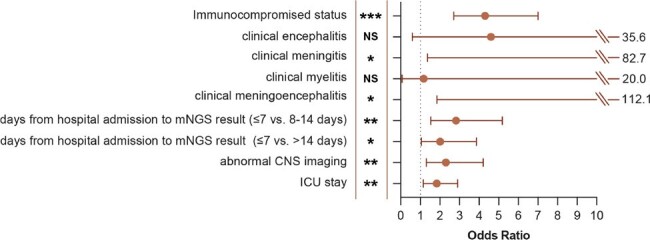
Significant clinical factors associated with mNGS positivity selected by multivariate analysis. The odds ratio and associated confidence intervals are plotted for each row (clinical factors). *, P < 0.05; **, P < 0.01; ***, P < 0.001; NS, non-significant.

**Results:**

Of 1,164 mNGS tests, 133 were positive for CNS infection-causing pathogens. By univariate analysis **(Table 1),** factors including hospitalization (*P* = 0.009), intensive care unit (ICU) admission (*P* = 0.02), immunosuppressed status (*P* < 0.0001), clinical meningitis or meningoencephalitis (*P* < 0.0001), and abnormal CNS imaging (*P* = 0.004) were significantly associated with a positive mNGS result. In contrast, demographic variables, including age, sex, race, and ethnicity, were not. For CSF, early mNGS testing performed by hospital day 7 (*P* < 0.002), elevated protein (*P* = 0.005), and low glucose (*P* = 0.001) were associated with a positive mNGS result, whereas the presence of red blood cells (P = 0.77), pleocytosis (*P* = 0.17), and neutrophil or lymphocyte predominance (*P* = 0.24) were not. Significant factors selected by multivariate analysis include ICU stay (OR = 1.8, 95%CI[1.2, 1.9]), acute abnormalities on imaging (OR = 2.3, 95%CI[1.3, 4.2]), early mNGS testing within 7 days of admission (OR = 2.8, 95%CI[1.5, 5.2]), meningitis or meningoencephalitis (OR = 10.7, 95%CI[1.4, 82.7] and OR = 14.4, 95%CI[1.9, 112.1]), and immunosuppressed status (OR = 4.3 95%CI[2.7, 7.0]) **(Figure 1)**.

**Conclusion:**

This study identified key factors for predicting positive CSF mNGS results that will be incorporated into a predictive scoring system to identify clinical scenarios for which testing is clinically indicated to maximize diagnostic yield. Real-time scoring metric will enhance diagnostic stewardship and optimize the cost-effectiveness of CSF mNGS testing.

**Disclosures:**

**Charles Chiu, MD, PhD**, Abbott Laboratories, Inc: Grant/Research Support|Biomeme: Advisor/Consultant|Biomeme: Board Member|BiomeSense: Advisor/Consultant|BiomeSense: Board Member|Delve Bio: Advisor/Consultant|Delve Bio: Board Member|Delve Bio: Grant/Research Support|Flightpath Biosciences: Advisor/Consultant|Flightpath Biosciences: Board Member|Mammoth Biosciences: Advisor/Consultant|Mammoth Biosciences: Board Member|Pathogen detection using next generation sequencing: US patent 11380421|Poppy Health: Advisor/Consultant|Poppy Health: Board Member

